# Patients’ Preferences for Artificial Intelligence Applications Versus Clinicians in Disease Diagnosis During the SARS-CoV-2 Pandemic in China: Discrete Choice Experiment

**DOI:** 10.2196/22841

**Published:** 2021-02-23

**Authors:** Taoran Liu, Winghei Tsang, Fengqiu Huang, Oi Ying Lau, Yanhui Chen, Jie Sheng, Yiwei Guo, Babatunde Akinwunmi, Casper JP Zhang, Wai-Kit Ming

**Affiliations:** 1 Department of Public Health and Preventive Medicine School of Medicine Jinan University Guangzhou China; 2 Faculty of Economics and Business University of Groningen Groningen Netherlands; 3 School of Finance and Business Shanghai Normal University Shanghai China; 4 Department of Obstetrics and Gynecology Brigham and Women’s Hospital Boston, MA United States; 5 Center for Genomic Medicine, Massachusetts General Hospital Harvard Medical School Harvard University Boston, MA United States; 6 School of Public Health The University of Hong Kong Hong Kong China (Hong Kong)

**Keywords:** discrete choice experiment, artificial intelligence, patient preference, multinomial logit analysis, questionnaire, latent-class conditional logit, app, human clinicians, diagnosis, COVID-19, China

## Abstract

**Background:**

Misdiagnosis, arbitrary charges, annoying queues, and clinic waiting times among others are long-standing phenomena in the medical industry across the world. These factors can contribute to patient anxiety about misdiagnosis by clinicians. However, with the increasing growth in use of big data in biomedical and health care communities, the performance of artificial intelligence (Al) techniques of diagnosis is improving and can help avoid medical practice errors, including under the current circumstance of COVID-19.

**Objective:**

This study aims to visualize and measure patients’ heterogeneous preferences from various angles of AI diagnosis versus clinicians in the context of the COVID-19 epidemic in China. We also aim to illustrate the different decision-making factors of the latent class of a discrete choice experiment (DCE) and prospects for the application of AI techniques in judgment and management during the pandemic of SARS-CoV-2 and in the future.

**Methods:**

A DCE approach was the main analysis method applied in this paper. Attributes from different dimensions were hypothesized: diagnostic method, outpatient waiting time, diagnosis time, accuracy, follow-up after diagnosis, and diagnostic expense. After that, a questionnaire is formed. With collected data from the DCE questionnaire, we apply Sawtooth software to construct a generalized multinomial logit (GMNL) model, mixed logit model, and latent class model with the data sets. Moreover, we calculate the variables’ coefficients, standard error, *P* value, and odds ratio (OR) and form a utility report to present the importance and weighted percentage of attributes.

**Results:**

A total of 55.8% of the respondents (428 out of 767) opted for AI diagnosis regardless of the description of the clinicians. In the GMNL model, we found that people prefer the 100% accuracy level the most (OR 4.548, 95% CI 4.048-5.110, *P*<.001). For the latent class model, the most acceptable model consists of 3 latent classes of respondents. The attributes with the most substantial effects and highest percentage weights are the accuracy (39.29% in general) and expense of diagnosis (21.69% in general), especially the preferences for the diagnosis “accuracy” attribute, which is constant across classes. For class 1 and class 3, people prefer the AI + clinicians method (class 1: OR 1.247, 95% CI 1.036-1.463, *P*<.001; class 3: OR 1.958, 95% CI 1.769-2.167, *P*<.001). For class 2, people prefer the AI method (OR 1.546, 95% CI 0.883-2.707, *P*=.37). The OR of levels of attributes increases with the increase of accuracy across all classes.

**Conclusions:**

Latent class analysis was prominent and useful in quantifying preferences for attributes of diagnosis choice. People’s preferences for the “accuracy” and “diagnostic expenses” attributes are palpable. AI will have a potential market. However, accuracy and diagnosis expenses need to be taken into consideration.

## Introduction

The phenomenon of uneven allocation and distribution of high-quality doctor resources has existed for centuries with the history of the modern medical industry, which brings about a series of problems such as gaps in diagnosis accuracy, speed, or accessibility in rural areas. According to a recent study, over 12 million patients in the United States have experienced one or more misdiagnoses, and the misdiagnosis rate is 5.08% [1]. Some developing countries still face the problem of scarcity of doctors. The World Health Organization (WHO) has suggested that 2.5 doctors per 1000 people are needed to guarantee primary health care [2]. However, it has been reported that there were only 1.9 doctors per 1000 people in 2017 in China, and 45% of WHO member countries still have less than 1 doctor per 1000 people [3]. Therefore, new medical technology, such as artificial intelligence (AI) technologies, urgently needs to be improved.

The recent outbreak of an epidemic caused by severe acute respiratory syndrome coronavirus 2 (SARS-CoV-2) is a severe threat to public health. The COVID-19 pandemic, with lockdowns and unprecedented restrictions on movement, brings a newer angle to the importance of AI diagnosis. With big data growth in biomedical and health care communities, AI is increasingly applied to antiepidemic medical practice.

Medical AI can be classified into eight main fields: medical imaging and diagnosis, medical research, medical risk analysis, drug mining, virtual nurse assistant, prognostics and health management, mental health, and nutrition [4,5]. AI diagnosis and treatment technologies have become increasingly mature and are expected to become mainstream. As far as we know, there is no clear proof of how health outcomes or costs interdepend and correlate [6-8], although AI diagnosis was assumed certainly to be the higher cost-performance option from a medical angle. Such a lack of proof of clear superiority of either diagnosis method gives prominence to the patients’ and medical institutions’ preferences. Thus, the acceptability may be enhanced by choosing and adapting a diagnostic program to cater to patients’ preferences.

In addition, the Ministry of Industry and Information Technology of China demanded an increase in the praxis of artificial intelligence in the precise prevention and control of epidemics [9]. AI algorithms combine chest computed tomography imaging reports with clinical symptoms, medical history, and laboratory examination to rapidly diagnose patients as infected by SARS-CoV-2. The AI system slightly reduced misdiagnosis by radiologists [10]. The COVID-19 detection neural network, a deep learning model, can precisely detect SARS-CoV-2 and distinguish it from other pneumonia [11]. Artificial neural network modeling of SARS-CoV-2 morbidity across the United States illustrated that a single-hidden-layer multilayer perceptron could interpret nearly 65% of the correlation with ground truth for the prognostication [12].

A few studies [13,14] have focused on the effect of outpatient waiting time, diagnosis time, follow-up after diagnosis, etc in patients’ decision making and concluded that these factors play a vital role in patients’ trade-off and relative policy making. However, with the development of AI in medicine and rise of AI diagnosis, patients start shifting their focus to the accuracy and expense of AI diagnosis. Thus, we aim to fill the gap that exists because almost no studies focus on the effects of accuracy or other attributes of AI diagnosis and clinicians in patients’ choice.

The objectives of this paper are to measure the extent of patients’ preferences for a range of characteristics of an AI diagnosis scheme in China and to determine what characteristics are more attractive and make AI a better alternative to defeat traditional medical methods. A technique that is in common use for visualizing preferences is the discrete choice experiment (DCE), in which different alternatives with various attributes are given in the form of a questionnaire to people who are invited to choose options. In this paper, we will construct and make a comparison of these three models: mixed logit (MXL) model, generalized multinomial logit (GMNL) model, and latent class model (LCM). Moreover, the importance of attribute levels and preference heterogeneity must be compared and reported when people are considering any AI diagnosis service.

## Methods

### Overview

We designed multiple choices in different scenarios consisting of 6 different randomly selected attributes using conjoint-related techniques. Questionnaires were created by Sawtooth software’s Lighthouse Studio modules (version 9.8.1) for general interviews and choice-based conjoint (CBC) scenario design. Respondents were aged 18 to 85 years. Meanwhile, in this data analysis section, we aimed to visualize and measure the percentage weight and importance of different attributes with selected models. From the perspective of public health, McFadden’s conditional logit [15], which is also known as multinomial logit (MNL) [16], is widely applied to organize, analyze, and predict the data we had and help with further analysis of statistical significance. However, the foundation of using this model is that we acquiesce that unobserved heterogeneity of preference does not exist across respondents. Therefore, we then need to introduce a MXL model [17] and a GMNL model [18], both of which take unobserved heterogeneity of preference into account. The LCM [19] might also be an appropriate model to apply here, since it divides the data into various groups with fixed segmented size and the evident probability of latent membership [20]. With different latent classes, we can clearly distinguish the most important attributes or attributes’ levels of each class and summarize these attributes with a significant percentage weight.

### Principle of DCE

Random utility theory [21] is the basic principle of DCE. The principle assumes that all the choice selectors have *M* different choice alternatives, and each choice alternative corresponds to a utility *W*. The utility *W* is consistent with a combination of fixed and random utility. The stationary utility *U* can be explained by some observable elements *x*, while the random factor *ε* represents the influence and interference unobserved utility and possible error. The goal of choice selectors is to choose the best combination with the supreme utility; then the probability of each combination alternative being selected can be expressed as a function of its fixed utility: *P = F(U)*. The specific form of the function depends on the distribution of random effects. In most model settings, the utility that is optical, *U_v_*, will be expressed as a linear combination of elements *x*, that is, *U_v_ = βx*. *β* is a coefficient, and its value and significance level can be estimated from the observation data.

### Selection of Attributes

Based on the relevant literature [22-24], we have assumed that the patients’ preference or satisfaction with the medical choices mainly depends on some specific features that make up the essential attributes of our experiment. Moreover, we performed a pilot test to get the attributes and levels that we need in our research. Patients in the outpatient queue of the First Affiliated Hospital of Jinan University (Guangzhou Overseas Chinese Hospital) and the First Affiliated Hospital of Sun Yat-sen University were interviewed and invited to have a discussion of what attributes patients are most likely to attach importance to. In addition, the possible attributes’ levels are hypothesized and set in a certain sequence in our questionnaire; for instance, accuracy ranks from 0% to 100%.

Therefore, in our questionnaire, six attributes have been contained for our experiment: (1) diagnostic method; (2) outpatient waiting time before being asked; (3) diagnosis time; (4) accuracy (ratio of correct diagnosis); (5) follow-up after diagnosis (whether the outpatient doctor/AI doctor can follow up and follow up at any time); and (6) diagnostic expenses. Every attribute and its levels are presented in Table 1.

**Table 1:** 6 different attributes hypothesized and their levels in discrete choice experiment questionnaire.

Diagnosis methods	Levels
Diagnostic methods	Clinicians’ diagnosis; AI diagnosis + clinicians’ confirmation; AI diagnosis
Outpatient waiting time	0 min; 20 min; 40 min; 60 min; 80 min; 100 min
Diagnosis time	0 min; 15 min; 30 min
Accuracy	60%; 70%; 80%; 90%; 100%
Follow-up after diagnosis	Yes; No
Diagnostic expenses^a^	¥0; ¥50; ¥100; ¥150; ¥200; ¥250

^a^A currency exchange rate of ¥1=US $0.15 is applicable.

### Questionnaire and DCE Design

The questionnaire contains two sections. In the first section, which is also known as demographic questions, we aim to allocate the respondents’ basic information: age, gender, and highest education level. In the second section, we use the CBC function in Sawtooth software to create various combinations of scenarios for respondents to choose from.

When we use the factorials method [25] to analyze the attributes to give the combinations of scenarios, we encounter several obstacles. Since we have 6 attributes that give 3240 (3×6×3×5×2×6) possibilities, we assume that we have 6 random questions and 100 sets, which also gives 600 different combinations. The difficulty is how to extract 600 representative combinations from 3240 combinations and obey two basic principles [26] at the same time: (1) balance and (2) orthogonality. Balance means each attribute level appears equally often within an attribute, and level 1 in attribute 1 equals level 2 in attribute 2. Orthogonality means each pair of levels appears equally often across all pairs of attributes. However, it is almost unrealistic to handle such a huge task. Therefore, we use Sawtooth software to help us select suitable combinations. We set six random tasks, one fixed task, and two concepts per task (excluding “None Option”). Meanwhile, we set the sample size 500 and assume that 5% of respondents would choose “None Option” and finally design the test.

The standard error of almost all the attributes’ levels is <0.05. Since the expense is a continuous variable of which the standard error could be slightly higher than 0.05, a sample size of 500 should be sufficient for our experiment. One of the CBC tasks of the DCE questionnaire has been presented in Table 2.

**Table 2:** An example scenario of choice-based conjoint in the questionnaire.

Attributes	Doctor A	Doctor B	None
Diagnostic methods	AI diagnosis	Clinicians' diagnosis	
Outpatient waiting time (min)	0	20	
Accuracy (%)	80	60	
Follow-up after diagnosis	No	Yes	
Diagnostic expenses (¥)^a^	150	200	
Which method would you choose?	Choose Doctor A	Choose Doctor B	No choice

^a^A currency exchange rate of ¥1=US $0.15 is applicable.

### Data Collection Procedure

We sent our website link containing our DCE questionnaire through social media apps such as Facebook and WeChat. In addition, we gave respondents some rewards such as Mi bands or a small cash payment if the respondents could fully complete the questionnaire.

### Statistical Analysis

#### GMNL Model and MXL Model

With collected data, some models can be applied in our analysis; first is the GMNL model. We do not use the MXL model or the scaled-multinomial logit (S-MNL) model here since the GMNL model, which is developed by Fiebig (2010) [27], nested a MXL model and scaled multinomial model. Meanwhile, the GMNL model could accurately describe consumers’ preferences and heterogeneity. According to Fiebig et al, the probability of respondent i choosing alternative j in choice situation t is given as follows:


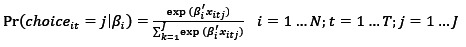


Here, *β_i_* is a vector of an individual-specific parameter and the individual coefficients of independent variables can be described as follows:

βi = σiβ + {γ + σi(1 – γ)}ηi

where the *β* is a constant vector, the “effect” *σ*_i_ in our model is a parameter of individual-specific scale, and *γ* is a parameter that decides how *σ_i_* and *η_i_* are different in some degrees. With this equation, when *σ_i_* equals 1, our GMNL model will become an MXL model. Meanwhile, when the variance of *η_i_* becomes 0, our GMNL model will turn into a S-MNL model. When *σ_i_* equals 1 and variance of *η_i_* equals 0 are both satisfied, the GMNL model will transform into an MNL model. Sawtooth will be needed to help run the coefficients of all attributes, standard errors, and *t* ratios to calculate *P* values. Differences between attributes are also needed to calculate the odds ratios. We calculate the odds ratio using the following equation:

odds ratio = exp (current effect – reference effect)

#### LCM

In addition, the LCM will be applied. LCM is a latent variable model because the latent variable is discrete. According to Greene and Hensher (2003) [28], the principle of LCM is that the observable attributes and latent heterogeneity decide the individual behavior. The heterogeneity changes with the unobserved factors. This model is used to sort individuals into a set of classes with a certain segmented size and scale, and different effects of each class have been estimated for different attributes. As well, the LCM will help us measure the differences and similarities of preference across classes of respondents. Attributes’ importance and part worth utilities will also be needed for visual analysis and comparison of attributes and deciding which attribute is the most essential from the people’s perspective. Additionally, the average maximum membership probability will help predict the certainty of the class into which respondents are divided.

## Results

### Respondents

428 participants (aged 18-85 years) who provided complete data were included in the analysis. Among those with complete data, 206 (48.1%) were male and 222 (51.9%) were female, while 2 of them were pregnant women.

### Attributes’ Levels and Utility Report

The average utility values of all the attributes’ levels were measured using the Utility Scaling Method with zero-centered differences. The highest utility levels of the six hypothesized attributes are “AI diagnosis + clinicians’ confirmation” (of “diagnosis method”), “20 min” (of “outpatient waiting time”), “15 min” (of “diagnosis time”), “100%” (of “accuracy”), “Yes” (of “follow-up”), and “¥0” (of “diagnostic expenses”), respectively. The most important attribute is “accuracy,” meaning that the majority of respondents ranked that attribute (Table 3).

**Table 3:** The utility report of different attributes’ levels.

Attributes and levels		Utility
**Diagnostic methods**		
	Clinician	−11.51
	AI + clinician	57.64
	AI	−46.13
**Outpatient waiting time (min)**		
	0	12.57
	20	35.41
	40	4.41
	60	−27.99
	80	−24.40
**Diagnosis time (min)**		
	0	−7.02
	15	4.14
	30	2.88
**Accuracy (%)**		
	60	−116.31
	70	−60.65
	80	−2.24
	90	59.75
	100	119.44
**Follow-up after diagnosis**		
	Yes	27.88
	No	−27.88
**Diagnostic expenses (¥)^a^**		
	0	47.91
	50	32.93
	100	32.25
	150	−5.92
	200	−24.91
	250	−82.25
**None**		
	N/A^b^	−235.59

^a^A currency exchange rate of ¥1=US $0.15 is applicable.

^b^N/A: not applicable.

### Logit Result of DCE and Attributes’ Percentage Importance

**Figure 1:**
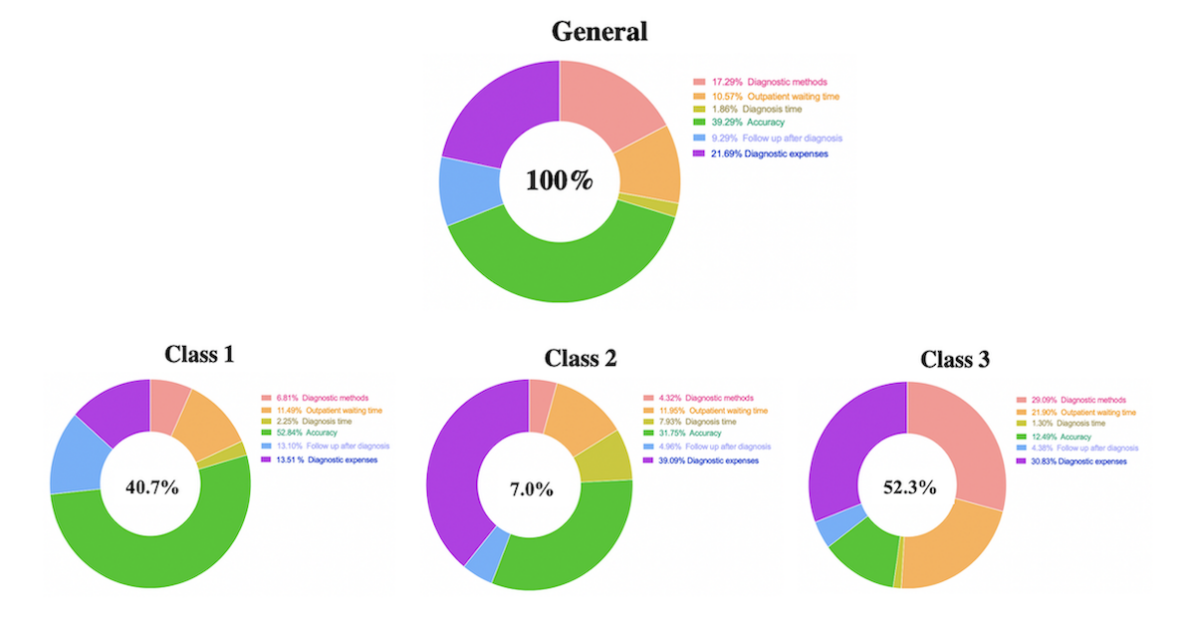
Percentage importance of attributes in general condition and latent class condition.

In general, it is obvious that attribute “accuracy” was the most important factor and most preferred when facing the diagnosis. As is presented in Figure 1, the percentage importance of attribute “accuracy” was 39.29%, which undoubtedly shows the position of accuracy of diagnosis in people’s minds. Attributes “diagnostic expenses” and “diagnostic method” ranked second and third, respectively.

The results of the logit analysis of all attributes’ levels are presented in Table 4. For the diagnosis method, the coefficient of level “AI + clinician” is positive, which means that the level “AI + clinician” is positively correlated with people’s preference and utility. In addition, the coefficients of levels “0 min,” “20 min,” and “40 min” (of “outpatient waiting time”) are positive, and others are negative. It is clear that people have a preference for shorter outpatient waiting time. For other attributes’ levels, people prefer to choose the higher accuracy level, greater possibility to follow up after diagnosis, and lower diagnosis expense. However, for the attribute “diagnosis time,” people unexpectedly prefer a longer diagnosis time rather than the “0 min” option.

**Table 4:** The result of logit analysis of preference in general (N=428).

Attributes and levels		Coefficient	Standard Error	*P* value	Odds ratio	95% CI
**Diagnostic methods**						
	Clinician	−0.07226	0.03829	.06	Reference	
	AI + clinician	0.37192	0.0386	<.001	1.559	(1.446-1.682)
	AI	−0.29966	0.03926	<.001	0.797	(0.738-0.860)
**Outpatient waiting time (min)**						
	0	0.09507	0.05818	.10	Reference	
	20	0.22298	0.05815	<.001	1.136	(1.014-1.274)
	40	0.03253	0.05965	.59	0.939	(0.836-1.056)
	60	−0.18624	0.0586	.002	0.755	(0.673-0.847)
	80	−0.16435	0.05915	.006	0.771	(0.687-0.866)
**Diagnosis time (min)**						
	0	−0.04446	0.03857	.25	Reference	
	15	0.03001	0.03853	.44	1.077	(0.999-1.162)
	30	0.01444	0.03831	.71	1.061	(0.984-1.143)
**Accuracy (%)**						
	60	−0.74382	0.06294	<.001	Reference	
	70	−0.4016	0.05957	<.001	1.408	(1.253-1.582)
	80	−0.0097	0.057	.87	2.084	(1.863-2.330)
	90	0.38431	0.05787	<.001	3.090	(2.759-3.461)
	100	0.77081	0.05943	<.001	4.548	(4.048-5.110)
**Follow-up after diagnosis**						
	Yes	0.18169	0.02415	<.001		
	No	−0.18169	0.02415	<.001		
**Diagnostic expenses (¥)^a^**						
	0	0.30678	0.06632	<.001	Reference	
	50	0.22572	0.06606	<.001	0.922	(0.810-1.050)
	100	0.20776	0.06673	.002	0.906	(0.795-1.032)
	150	−0.04055	0.06692	.55	0.707	(0.620-0.806)
	200	−0.16992	0.06693	.01	0.621	(0.545-0.708)
	250	−0.52978	0.06916	<.001	0.433	(0.378-0.496)

^a^A currency exchange rate of ¥1=US $0.15 is applicable.

For the *P* value of these attributes’ levels, we assume that if the *P* value of a level is less than .05, then this level is statistically significant; when the *P* value of a level is less than .001, then this level is extremely statistically significant. We found that “AI + clinician” and “AI diagnosis” for “diagnosis methods” are extremely statistically significant; “20 min” of outpatient waiting time is extremely statistically significant; “60 min” and “80 min” are statistically significant; “60%,” “70%,” “90%,” “100%” of “accuracy” are extremely statistically significant; both levels of attribute “follow up after diagnosis” are extremely statistically significant. “¥0’” of attribute “diagnostic expenses” is extremely statistically significant, and all the other levels of diagnostic expenses are statistically significant.

The odds ratio is a commonly used indicator in case-control epidemiological studies. In our analysis and calculation results (Table 4), we find that some odds ratios of the attributes’ levels compared to the reference level are greater than one, which means that the probability of people’s choosing of these levels is higher than the previous one or the reference. Taking the level “Clinician” of attribute “diagnostic methods” as the reference, level “AI + clinician” has an odds ratio of 1.559 (95% CI 1.446-1.682). The odds ratio of level “20 min” (of “outpatient waiting time”) is 1.136 (95% CI 1.014-1.274) with the reference of level “0 min.” Levels “15 min” and “30 min” (of “diagnosis time”) have odds ratios 1.077 and 1.067, respectively, with the reference of level “0 min” (95% CI 0.999-1.162 and 0.984-1.143, respectively). All the levels of the attribute “accuracy” were greater than one, which means the preference weights increase with the accuracy. Meanwhile, all the odds ratios of expense compared to the reference are smaller than zero, which refers to a preference of “free diagnosis” for the majority of people.

### Latent Class Analysis Result

We compared these potential models and selected the model that maximized the area under the receiver operating characteristic curve and minimized the Akaike information criterion (AIC) [29,30] and Bayesian information criterion (BIC) [31] to penalize for model complexity. According to AIC, 5 classes should be the best choice for our model. However, if we must choose with BIC, then a 2-class option should be the most appropriate one, since 2-class has the lowest BIC. Under such circumstances, we compare ABIC, which means sample size–adjusted BIC [32] and involves sample size value. After comparing, the 3-class option has the lowest value of ABIC. Therefore, the most suitable number of latent classes in our model was 3 (Table 5 and 6). First of all, we divided all 428 respondents into 3 classes with segment sizes of 174 (40.7%), 30 (7.0%), and 224 (52.3%). The average maximum membership probability is around 0.87 and the percent certainty was 35.30, which is relatively low, meaning that there was not much uncertainty according to the respondents divided into classes.

**Figure 2:**
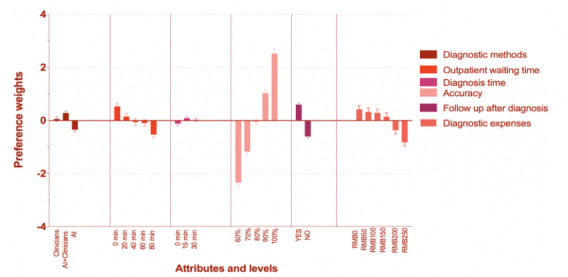
Latent class percentage weights in class 1. AI: artificial intelligence; RMB: yuan renminbi.

For class 1, *t* ratios for attributes “diagnostic expenses,” “‘accuracy,” and “follow-up after diagnosis” were significant across all treatment modalities. Attribute “accuracy” was the most important factor for patients with 52.8%, followed by “diagnostic expenses” and “follow-up after diagnosis” with percentage importance 13.51% and 13.10%, respectively (Figure 1). Meanwhile, the span of percentage weights of “accuracy” is obvious (Figure 2), from −2.33 to 2.52. Meanwhile, the span of attribute “diagnostic expenses” is from −0.819 to 0.423. The preference weights of “diagnostic expenses” decrease with the increasing of the expenses. The preference weight of attribute “follow-up after diagnosis” is from −0.602 to 0.602. This is −0.602 for “No follow-up” and 0.602 for “follow-up”, which is symmetrical. In addition, the odds ratio of level “AI + clinicians” was 1.247 (95% CI 1.036-1.463), meaning that the majority of patients prefer durable treatments over a single treatment. As well, all the levels of attribute “diagnosis time” with the reference level “0 min” are larger than one. At the same time, the odds ratio (Table 7) of the levels of attribute “accuracy” increases with the accuracy rate, meaning that people’s preference weight increases with the accuracy.

**Table 5:** Result of 3 latent classes’ conditional logit analysis.

Attributes and levels		Class 1, n=174 (40.7%)			Class 2, n=30 (7.0%)			Class 3, n=224 (52.3%)		
		Coefficient	SE	*P* value	Coefficient	SE	*P* value	Coefficient	SE	*P* value
**Diagnostic methods**										
	Clinician	0.062	0.083	.046	−0.175	0.282	.54	−0.153	0.050	.003
	AI + clinician	0.282	0.082	<.001	−0.085	0.287	.77	0.518	0.052	<.001
	AI	−0.344	0.085	<.001	0.260	0.286	.37	−0.365	0.051	<.001
**Outpatient waiting time (min)**										
	0	0.530	0.128	<.001	−0.385	0.476	.43	−0.028	0.077	.72
	20	0.147	0.124	.24	−0.128	0.422	.76	0.351	0.079	<.001
	40	−0.057	0.132	.67	0.819	0.365	.03	0.091	0.080	.25
	60	−0.095	0.127	.45	−0.330	0.455	.48	−0.314	0.079	<.001
	80	−0.526	0.122	<.001	0.023	0.414	.96	−0.100	0.079	.20
**Diagnosis time (min)**										
	0	−0.117	0.084	.17	0.317	0.280	.27	−0.021	0.051	.68
	15	0.089	0.082	.28	−0.481	0.345	.17	0.018	0.051	.72
	30	0.028	0.082	.73	0.164	0.280	.56	0.003	0.051	.95
**Accuracy (%)**										
	60	−2.337	0.166	<.001	−1.717	0.803	.04	−0.209	0.080	.01
	70	−1.170	0.131	<.001	−0.693	0.555	.22	−0.129	0.078	.10
	80	−0.050	0.112	.65	0.353	0.457	.45	0.034	0.078	.67
	90	1.036	0.122	<.001	0.577	0.406	.17	0.135	0.079	.09
	100	2.522	0.169	<.001	1.480	0.370	<.001	0.170	0.079	.03
**Follow-up after diagnosis**										
	Yes	0.603	0.059	.003	0.250	0.207	.24	0.066	0.031	.04
	No	−0.603	0.059	.03	−0.250	0.207	.24	−0.066	0.031	.035
**Diagnostic expenses (¥)^a^**										
	0	0.424	0.140	.003	0.313	0.492	.53	0.398	0.090	<.001
	50	0.324	0.143	.03	1.831	0.412	<.001	0.131	0.088	.14
	100	0.289	0.142	.04	−0.228	0.506	.66	0.236	0.090	.009
	150	0.144	0.149	.33	0.284	0.460	.54	−0.102	0.089	.25
	200	−0.361	0.146	.01	−2.106	0.983	.04	−0.123	0.089	.17
	250	−0.819	0.144	<.001	−0.093	0.533	.86	−0.538	0.091	<.001

^a^A currency exchange rate of ¥1=US $0.15 is applicable.

**Table 6:** Percent certainty and information criteria for model with 3 latent classes.

Characteristic	Value
Certainty (%)	35.307
Akaike information criterion	3580.631
Bayesian information criterion	3922.719
Sample size–adjusted Bayesian information criterion	3735.263

**Table 7:** The odds ratios and confidence intervals of attributes’ levels in 3 classes.

Attributes and levels		Class 1, n=174 (40.7%)		Class 2, n=30 (7.0%)		Class 3, n=224 (52.3%)	
		OR^a^	95% CI	OR	95% CI	OR	95% CI
**Diagnostic methods**							
	Clinician	Reference	N/A^b^	Reference	N/A	Reference	N/A
	AI + clinician	1.247	(1.036-1.463)	1.094	(0.624-1.920)	1.958	(1.769-2.167)
	AI	0.666	(0.564-0.787)	1.546	(0.883-2.707)	0.809	(0.732-0.895)
**Outpatient waiting time (min)**							
	0	Reference	N/A	Reference	N/A	Reference	N/A
	20	0.681	(0.535-0.868)	1.293	(0.566-2.957)	1.460	(1.252-1.703)
	40	0.556	(0.429-0.720)	3.332	(1.628-6.821)	1.126	(0.963-1.316)
	60	0.535	(0.418-0.686)	1.057	(0.433-2.580)	0.751	(0.643-0.877)
	80	0.348	(0.274-0.442)	1.504	(0.688-3.388)	0.930	(0.797-1.085)
**Diagnosis time (min)**							
	0	Reference	N/A	Reference	N/A	Reference	N/A
	15	1.229	(1.047-1.444)	0.450	(2.229-0.885)	1.040	(0.942-1.149)
	30	1.156	(0.986-1.357)	0.858	(0.469-1.485)	1.024	(0.927-1.132)
**Accuracy (%)**							
	60	Reference	N/A	Reference	N/A	Reference	N/A
	70	3.214	(2.484-4.159)	2.785	(0.938-8.271)	1.084	(0.930-1.263)
	80	9.849	(7.912-12.258)	7.931	(3.240-19.417)	1.275	(1.095-1.485)
	90	29.173	(22.962-37.064)	9.920	(4.480-21.962)	1.411	(1.207-1.648)
**Follow-up after diagnosis**							
	Yes	Reference	N/A	Reference	N/A	Reference	N/A
	No	0.300	(0.267-0.337)	0.607	(0.405-0.910)	0.876	(0.824-0.931)
**Diagnostic expenses (¥)^c^**							
	0	Reference	N/A	Reference	N/A	Reference	N/A
	50	0.905	(0.683-1.199)	4.563	(2.037-10.222)	0.766	(0.644-0.911)
	100	0.847	(0.662-1.154)	0.583	(0.216-1.571)	0.851	(0.713-1.015)
	150	0.756	(0.565-1.102)	0.972	(0.394-2.394)	0.606	(0.509-0.722)
	200	0.456	(0.343-0.607)	0.089	(0.013-0.612)	0.594	(0.499-0.707)
	250	0.289	(0.217-0.383)	0.666	(0.234-1.895)	0.392	(0.328-0.469)

^a^OR: odds ratio.

^b^N/A: not applicable.

^c^A currency exchange rate of ¥1=US $0.15 is applicable.

**Figure 3:**
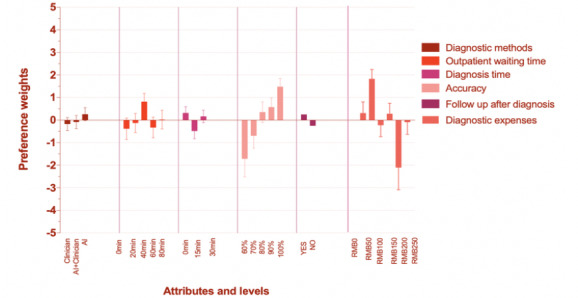
Latent class percentage weights in class 2. AI: artificial intelligence; RMB: yuan renminbi.

For class 2, the attribute “accuracy,” as well as “diagnostic expenses,” was relatively important. Meanwhile, “diagnostic expenses” was (surprisingly) the most important for respondents among the attributes with a percentage weight of 39.09%, followed by “accuracy” with a percentage of 31.75% (Figure 1). The span of the percentage weights of these two attributes has been presented in Figure 3. The percentage weights for attribute “accuracy” were from −1.717 to 1.480, and −2.10 to 1.830 for “diagnostic expenses” (Figure 3). From *P* values, we find that almost all the levels are not statistically significant except the “100%” level of “accuracy” and level “¥50” of “diagnostic expenses.” The odds ratio (Table 7) displays that the AI method is the best of three methods. The odds ratio of levels of “outpatient waiting time” are all greater than 1, and patients’ preference weight still increases with the increasing of “accuracy” as in class 1. For attribute “diagnostic expenses,” only the level “¥50” has an odds ratio greater than 1.

**Figure 4:**
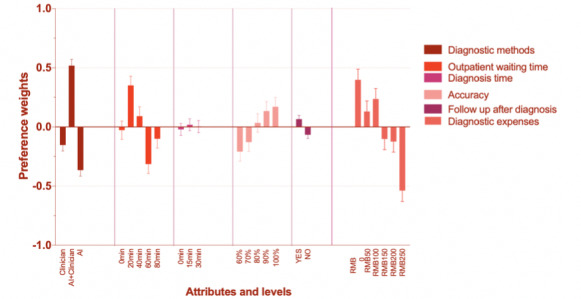
Latent class percentage weights in class 3. AI: artificial intelligence; RMB: yuan renminbi.

For class 3, attributes “diagnosis method” and “outpatient waiting time” were more statistically significant across all the attributes. For respondents in this class, the percentage importance of “diagnosis method” and “outpatient waiting time” were 29.09% and 30.83%, respectively (Figure 1). From Figure 4, we find that the span for diagnostic methods is from −0.364 to 0.518. Meanwhile, the span for diagnostic expenses is from −0.538 to 0.397. In addition, the odds ratio of level “AI + clinicians” of attribute “diagnostic method” was 1.985 (95% CI 1.769-2.167), which is greater than 1, meaning that respondents in class 3 also prefer durable diagnosis mode rather than single mode, similar to the condition of class 1. All of the odds ratios of levels of attributes “outpatient waiting time” and “accuracy” are greater than 1. Meanwhile, the odds ratio continues increasing with the increase of accuracy, the same condition of the previous two classes.

For these 3 latent classes, attribute “accuracy” is the most preferred factor in two classes (class 1 and class 2), while “diagnostic expenses” is the most preferred factor in class 3. In addition, the odds ratio of levels of attribute “accuracy” always increases with the increasing of accuracy rate, meaning that people’s preference will always grow with the increase, and higher accuracy is always preferred.

## Discussion

### Principal Findings

Several different models were used in our study to research people’s preference for various attributes, including a GMNL model and LCM; both models presented with their own merits and shortcomings.

For the GMNL analysis and the utility report, we found that “accuracy” was the most important thing for respondents and most of the levels of attributes “diagnostic methods” and “accuracy” were statistically significant. From the odds ratio, most of the respondents have a preference for the AI diagnosis plus clinicians’ confirmation with outpatient waiting time of 20 minutes, diagnosis time of 15 minutes, and 100% accuracy. We would expect that the outpatient waiting time of 0 minutes and diagnosis time of 0 minutes should be preferred. However, the preferred choice was not 0 minutes. We assume that some people believe that a longer diagnosis time can give them more credibility and a feeling of safety. Furthermore, the odds ratio of 1.559 for “AI diagnosis + clinicians’ confirmation” was evidently higher than only “AI diagnosis,” which reflects that, for now, the vast majority of people still cannot totally trust the AI diagnosis due to the factors of AI uncertainty. AI diagnosis acting in concert with clinicians will significantly guarantee the accuracy. Another defect of AI diagnosis is that patients follow up after diagnosis, which means that patients who cannot receive follow-up have no alternatives but to head to clinicians as outpatients, which indirectly results in missing the best patient treatment time. That also leads to huge inconvenience to both patients and clinicians. Those who have an evident preference for only AI diagnosis may as a result of freshness and relatively higher diagnosis time of AI method, with sacrifice of accuracy in their mind. Thus, we can still consider the clinicians’ diagnosis to remain irreplaceable, at least in the short term. The limitation of the GMNL model was also obvious; some respondents with preferences for other diagnostic methods and different diagnosis expenses cannot be reflected.

From the LCM, our study finds that respondents who are divided into 3 classes show different preferences and different patient profiles. There was slight heterogeneity compared to the GMNL model. Specifically, respondents in class 1 and class 2 still attach importance to “‘accuracy”; however, respondents in class 3 mostly pay attention to “diagnostic expenses.” Although medical insurance and accessibility are quite advanced today, some patients hardly get access to basic medical diagnosis or treatment, particularly in some underdeveloped or remote areas in China. Some old people would rely on their own self-healing function or immune system [33] rather than go to the hospital due to their outdated concepts of unaffordable diagnosis and treatment expenses. Therefore, the acceptability of AI diagnosis or even modern medical techniques among old people is palpably lower than among new generations in China due to the concepts of cost. In addition, we hypothesized that the numbers of hospitals in remote areas is undoubtedly lower than those in urban or advanced areas, which results in the people living in remote areas having to undertake the transportation time and cost. The long transit time and cost could also sometimes be fatal to these people since that would also force them to stay at home and miss the most appropriate diagnosis and treatment time. To sum up, the promotion and spread of AI diagnosis cannot ignore the need to set a suitable diagnosis price or give some discount and bonus according to the wealth status of patients.

Several previous studies [13] have found that most patients believe the outpatient waiting time plays a vital role in their decision-making behavior when faced with various choices of hospitals and clinicians. In addition, few studies [14] have attached importance to the quality and quantity of follow-up after clinical diagnosis. However, all of these studies ignored the effect of diagnosis accuracy and diagnosis expense in patients’ trade-offs. Especially in the era of artificial intelligence, there are few studies addressing the accuracy of AI versus human clinicians rather than the outpatient waiting time or other factors. With diagnostic accuracy, clinically, the AI system can be programmed to probe and mark out some cancer indications such as prostate cancer and is more accurate than experts [34]. The clinical application could reduce pathology workload in this epidemic and in future clinical work. An AI system with expert-level grading performance might contribute a second opinion, aid in standardizing grading, and provide expert advice in areas with poor sanitation. As cloud computing capabilities come closer to life, overcomes limited memory, and central processing unit power [35]. We believe ever more and more people will trust the rapid diagnostic capabilities of AI. There is a lot of SARS-CoV-2–related scientific research undertaken to use AI to combat this pandemic by deploying new methods in the development of vaccines and drugs, as well as for public awareness [36]. Relying on the advantages of AI for medical auxiliary diagnosis, image analysis, remote consultation, etc during the outbreak of COVID-19, many AI devices have been used in first-line medicine. Moreover, AI is reducing cross infection. It has played an important role in therapeutic innovation. Health QR Code, which is a fusion of AI and big data, is a mobile phone app for everyone and uses red, yellow, and green colors to provide simple and effective intelligent services for personnel communication and economic and trade exchanges in the China in the “postepidemic era.”

AI will continue to play an increasingly important role in controlling the public health crisis to save lives and economic recovery. AI contact tracing based on mobile communication technology becomes more mature [37]. The AI systems combining computed tomography and clinical symptoms can help to quickly diagnose SARS-CoV-2 patients [10]. A surrogate rapid diagnosis technique that is based on a deep learning neural network can be applied for discovering SARS-CoV-2 by analyzing the visual chest radiography imaging of patients [38].

### Limitations

#### Limitations for Our DCE

Theoretically, the larger the sample size is, the smaller the changes we will find in the DCE; however, due to various reasons related to the pandemic, we applied convenience sampling in the data collection procedure. Thus, our sample size is relatively small and underrepresentative. Furthermore, the other significant shortcoming of our DCE data is that our statistics do not stand for the point of view of all the people in China or other people worldwide due to the limited transmission of our questionnaire. We did not set the diseases as acute or chronic, which is an exogenous factor that will affect people's choice of waiting time.

#### Limitations for AI Diagnosis Propaganda

With AI diagnosis, there exists a requirement for rapid delivery and logistics of medicine. Furthermore, the conceptual propaganda for AI diagnosis and treatment are still not in place, particularly in some rural areas and among some old people with relatively traditional medical concepts. Confidence and trust in the AI diagnostic method still have a long way to go.

### Conclusion

Segments of patients’ preferences for these diagnosis options seem to be homogenous and convergent. All the attributes hypothesized and attributes’ levels are evidently not ignorable during the implementation and widespread use of AI diagnosis techniques. People’s preference for “accuracy” was obvious across different classes. Although “online treatment” has become more common today, accuracy has been sacrificed in exchange for so-called convenience, which is totally unwise. In addition, AI diagnosis technique developers as well as technology sellers, including hospitals, should take diagnosis expense into consideration and make pricing rules more flexible in the light of areas’ economic development and individual patients’ wealth status.

AI will definitely have a potential market and bright future, especially in the ongoing COVID-19 pandemic, since the AI diagnostic technology can ease the requirements of professional clinicians worldwide, particularly in rural areas.

## Abbreviations

ABIC: sample size–adjusted BIC
AI: artificial intelligence
AIC: Akaike information criterion
BIC: Bayesian information criterion
CBC: choice-based conjoint
DCE: discrete choice experiment
GMNL: generalized multinomial logit
LCM: latent class model
MXL: mixed logit
OR: odds ratio
S-MNL: scaled-multinomial logit
WHO: World Health Organization
